# Isolation and Characterization of a Porcine Getah Virus Strain from Sichuan Province

**DOI:** 10.3390/vetsci12030276

**Published:** 2025-03-15

**Authors:** Lina Shao, Mincai Nie, Baoling Liu, Fengqin Li, Tong Xu, Lei Xu, Lishuang Deng, Hanyu Li, Lei Zhao, Youyou Li, Leyi Zhang, Yixin Yan, Zhiwen Xu, Ling Zhu

**Affiliations:** 1 Key Laboratory of Animal Disease and Human Health of Sichuan Province, College of Veterinary Medicine, Sichuan Agricultural University, Chengdu 611130, China; shaolina30@126.com (L.S.); sicdynmc@163.com (M.N.); lbl09137648@163.com (B.L.); lfqsean@126.com (F.L.); xutong123456@126.com (T.X.); xu_lei2013@126.com (L.X.); 18428374864@163.com (L.D.); 17742462781@163.com (H.L.); 19140264512@163.com (L.Z.); sclyy@hotmail.com (Y.L.); zhangleyi210@163.com (L.Z.); 15223648325@163.com (Y.Y.); 2 China College of Animal Science, Xichang University, Xichang 615013, China; 3 Sichuan Key Laboratory of Animal Epidemic Disease and Human Health, College of Veterinary Medicine, Sichuan Agricultural University, Chengdu 611130, China

**Keywords:** Getah virus, phylogenetic analysis, isolation, vaccine development, E2 protein

## Abstract

Following its initial documented emergence in Hunan’s swine facilities in 2017, GETV has inflicted considerable financial damage upon China’s pork production sector. Beyond its impact on swine, GETV also poses a potential risk to other animal species and public health, primarily due to its mosquito-borne transmission. Between September 2020 and May 2021, a GETV outbreak occurred on a commercial swine farm in Sichuan Province, where affected animals exhibited movement disorders, neurological symptoms, and mild diarrhea, with severe cases resulting in mortality. To elucidate the genetic characteristics and transmission dynamics of the virus, a comprehensive investigation of this outbreak was conducted. Clinical samples from diseased or deceased swine were collected and analyzed by PCR, confirming the presence of GETV infection. Additionally, differential diagnostic assays were performed for other common swine pathogens, including porcine reproductive and respiratory syndrome virus (PRRSV), pseudorabies virus (PRV), classical swine fever virus (CSFV), and atypical porcine pestivirus (APPV). All tests yielded negative results, confirming GETV as the sole etiological agent of the outbreak. The GETV-positive samples were inoculated into BHK-21 cells, leading to the isolation of a GETV strain (SC202009). Whole-genome sequence analysis revealed that SC202009 belongs to the GIII genotype of GETV, with a nucleotide sequence identity ranging from 95.1% to 99.8% compared to reference sequences in GenBank. This high level of sequence identity suggests that SC202009 is closely related to other circulating strains in the region, indicating potential local transmission networks and endemic viral circulation. The nucleotide sequence identity of the *E2* gene ranged from 94.2% to 99.6%, while the E2 protein amino acid sequence identity ranged from 97.2% to 99.5%. Phylogenetic analysis further indicated that SC202009 is genetically distant from the original Malaysian prototype strain (MM2021) but shares high sequence identity with several strains isolated in Sichuan Province, including SC266, SC201807, and SC483. Notably, NMJA_F2_18-8L-NH-Cxp-Y-1-1 was isolated from *Culex pipiens*, suggesting a mosquito-borne transmission route. These findings provide novel insights into the regional dissemination patterns and genetic diversity of GETV in China. These results also highlight the importance of the continuous surveillance of viral genetic variations and the elucidation of transmission dynamics to develop effective control strategies and mitigate potential public health risks. Future studies should focus on the ecological and environmental factors influencing GETV transmission, particularly the role of mosquito vectors in cross-species viral spread, to enhance targeted prevention and control measures for this emerging arboviral threat.

## Simple Summary

This study successfully isolated a strain of the Getah virus (GETV) from clinical samples, designated as SC202009, and deposited its genome sequence in the GenBank database under accession number MK693225. The genetic and biological properties of this strain were analyzed for further characterization. The virus was classified within the GETV GIII group. Notably, the E2 protein sequence of the SC202009 strain was highly conserved. Additionally, SC202009 exhibited the typical structural features of alphaviruses. This virus is relatively easy to cultivate, reaching a maximum infection titer of 10^−9.125^ TCID_50_/0.1 mL, and induces significant cytopathic effects and cell death in BHK-21 cells. Moreover, the virus displayed good genetic stability in BHK-21 cells, making it a promising candidate for further research on GETV seed viruses and vaccine development. These findings provide valuable insights into GETV seed virus research and vaccine development.

## 1. Introduction

**Figure 1:**
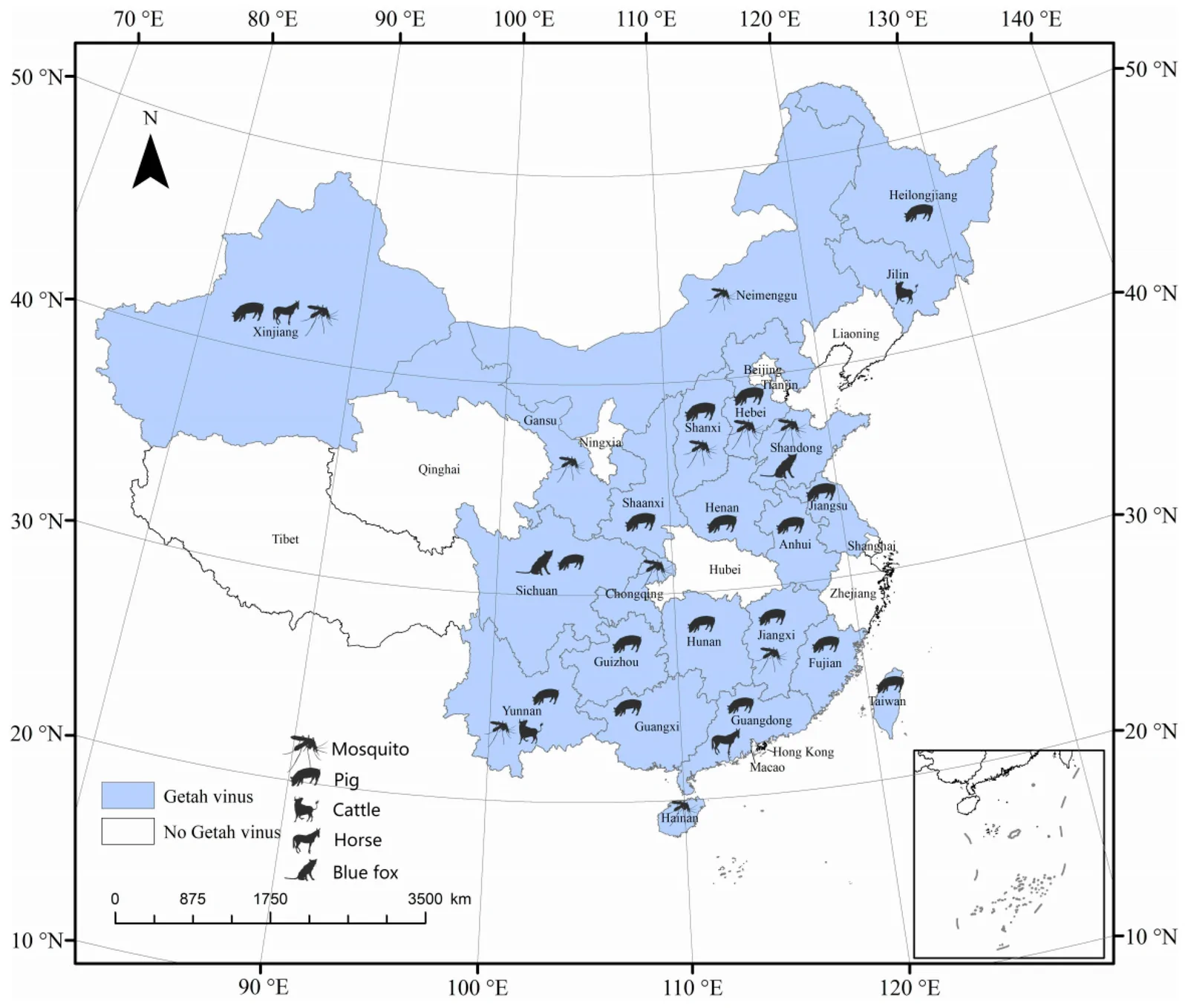
Geographical distribution of GETV in China, with animal host isolates marked using distinct animal patterns for each province.

Belonging to the *Alphavirus* genus within the family Togaviridae, GETV is classified as an arbovirus. These viral particles exhibit a spherical morphology, are enveloped, and feature surface projections, containing a single-stranded positive-sense RNA genome [1,2]. Following its initial isolation from Malaysian *Culex mosquitoes* in 1955, GETV has undergone extensive scientific investigation. The prototype strain MM2021 serves as a reference for understanding the virus’s genetic and biological characteristics. GETV circulates across approximately 13 countries and regions in the Pacific Rim and Eurasian continent [3], particularly in Asia, Australia, and parts of Europe, where it maintains transmission cycles primarily through mosquito species such as *Culex* and *Aedes* [4]. In China, GETV has been isolated from mosquitoes in several regions, including Inner Mongolia Autonomous Region, Yunnan Province, Xinjiang Autonomous Region, and Shanxi Province [5,6,7,8] (Figure 1). Notable strains such as LN0684 and YN12031 have been identified from *Armigeres subalbatus*. As a typical arbovirus, GETV efficiently replicates within arthropod vectors and utilizes them for transmission, with *Armigeres subalbatus* serving as a key vector species [9].

GETV has become endemic in various regions worldwide, leading to periodic outbreaks that result in significant economic losses for local livestock industries. Infections with GETV primarily affect pigs and horses. In pigs, clinical manifestations include reproductive disorders and neurological symptoms, while in horses, GETV typically causes severe rashes, fever, and edema [10,11,12]. Documented initial GETV infection episodes affecting equine and porcine populations have emerged across numerous nations, with notable epizootics among swine reported in regions such as India, South Korea, Thailand [13], mainland China, and other regions [14]. Following the first outbreak of porcine GETV in Hunan Province in 2017 [15], the virus has been identified in a diverse range of mammalian hosts, including horses, pigs, blue foxes, and cattle [16,17,18,19,20,21,22,23,24,25,26,27,28] (Figure 1). These findings suggest a continuous expansion of GETV’s host range and geographical distribution, indicating an increased risk of outbreaks across multiple species.

Despite increasing reports of GETV infections, our understanding of the genetic diversity, molecular characteristics, and epidemiology of circulating GETV strains in China remains limited. As one of the world’s largest swine producers, China faces unique challenges in controlling emerging infections like GETV, particularly given its high transmissibility and pathogenic potential in pigs. This study focused on the isolation and characterization of a porcine strain of GETV discovered in Sichuan Province, China. By addressing existing gaps in research regarding the genotyping and transmission of GETV within swine populations, this study highlights the genetic diversity of Sichuan GETV strains and the genetic stability of the virus. These findings provide a foundational basis for comprehending the transmission dynamics of GETV and contribute to vaccine development.

## 2. Materials and Methods

### 2.1. Sample Collection and Detection

Between September 2020 and May 2021, a disease outbreak occurred on a commercial pig farm in Sichuan Province, where affected animals exhibited movement disorders, neurological symptoms, and mild diarrhea, with severe cases resulting in mortality. The overall positivity rate for GETV was 16% (19/118), as previously reported by our laboratory [29]. Tissue samples, including samples from the spleen, lungs, kidneys, lymph nodes, and liver, were aseptically collected from the affected pigs and homogenized in phosphate-buffered saline (PBS). Total RNA extraction from tissue homogenate supernatants was performed utilizing TRIzol reagent (Invitrogen, Waltham, MA, USA), followed by cDNA synthesis through reverse transcription with thePrimeScript RT Reagent Kit according to the manufacturer’s protocols (Takara, Dalian, China). Samples that could not be processed immediately were stored at −80 °C for long-term preservation.

PCR amplification was performed using the Bio-Rad CFX96 PCR Detection System (Bio-Rad, Hercules, CA, USA), with a 10 µL reaction mixture consisting of 5 µL of 2× Taq PCR Master Mix (Vazyme, Nanjing, China), 0.5 µL (10 µM) of each primer, 1 µL of a viral cDNA template, and 3 µL of nuclease-free water. The thermal cycling conditions began with initial denaturation at 95 °C for 4 min, followed by 35 cycles of denaturation at 95 °C for 30 s, annealing at 56 °C for 10 s, and extension at 72° C for 5 min, with a final extension at 72 °C for 10 min [30]. Following amplification, PCR products were analyzed using 1% agarose gel electrophoresis. Primers specific to PRRSV, PRV, APPV, CSFV, and GETV were designed based on sequence data obtained from GenBank (see Supplementary Table S1).

### 2.2. Viral Growth and Cytopathic Effect Analysis

GETV-positive clinical samples were selected for subsequent virus isolation. Supernatants from these samples were filtered through a 0.22 μm filter (Millipore, Billerica, MA, USA), and 200 μL of the filtrate was inoculated onto confluent BHK-21 cells. The mixture was allowed to adsorb for 1 h at 37 °C. Following the adsorption phase, DMEM (Thermo Scientific, MA, USA) containing 2% FBS (Thermo Scientific, MA, USA) plus 1% dual-antibiotics (Beyotime, Shanghai, China) replaced, the original medium, with subsequent cell incubation conducted at 37 °C under 5% CO_2_ atmospheric conditions. Regular assessment of cellular conditions was performed. Upon observation of cytopathic effects (CPEs), the culture supernatants were harvested, and 200 μL was transferred to fresh confluent BHK-21 cells for virus passage. This passaging process was repeated three times. The presence of GETV in the cultures was confirmed by PCR, specifically targeting the *Cap* and *nsp3*.

DMEM was used to prepare 10-fold gradient dilutions of GETV SC202009. BHK-21 cells cultured in 96-well plates received 100 μL of virus dilutions ranging from 10^7^ to 10^13^, with each dilution tested across eight duplicate wells. The plates underwent incubation at 37 °C with a 5% CO_2_ atmosphere while being monitored for cytopathic alterations, and we subsequently determined TCID_50_ values through Reed–Muench methodology. This experiment was repeated in triplicate.

After diluting the virus to 10^3^ TCID_50_/0.1 mL in DMEM, it was added to 6-well plates containing BHK-21 cells (200 μL/well) and allowed to adsorb for 1 h at 37 °C. The medium was then replaced with DMEM containing 2% FBS. Following adsorption, fresh DMEM supplemented with 2% FBS replaced the inoculum, and plates were returned to the tissue culture environment for continued incubation. Both cells and supernatants were collected after 4, 8, 12, 24, 36, 48, 60, and 72 h. Cultures were assessed at each time point using the TCID_50_ method, which facilitated the construction of a one-step growth curve [31].

The viral suspension was added to BHK-21 cells at a density of 200 μL per well and allowed to adsorb at 37 °C for 1 h. The liquid was then replaced with DMEM containing 2% FBS. Following 12 h of growth at 37 °C in a 5% CO_2_ environment, the cells were harvested using trypsin. The culture supernatants were centrifuged and the supernatants were discarded. Subsequently, 1 mL of 0.5% glutaraldehyde (Beyotime, Shanghai, China).was added to fix the cells for 5 min. The culture supernatants were analyzed by electron microscopy. Concurrently, the cells in the 6-well plate were fixed with 4% paraformaldehyde for 15 min, permeabilized with 0.5% Triton X-100 (Beyotime, Shanghai, China) for 20 min, and blocked with 5% bovine serum albumin (diluted in PBS) for 60 min. Anti-Rabbit polyclonal antibody against the GETV E2 protein (1:200) (Self-prepared and stored in our laboratory)was incubated at 4 °C overnight, followed by five washes with PBST and incubation with Alexa Fluor 647-labeled Goat Anti-Rabbit IgG (1:1000) (Beyotime, Shanghai, China).for 60 min. After further washing with PBST, the cells were evaluated microscopically [32].

### 2.3. Complete GETV SC202009 Sequencing

Primers targeting complete gene segments were developed according to GETV SC201809 genomic data (MK693225) deposited in GenBank repository (details in Supplementary Table S2). PCR amplicons underwent purification via DiaSpin DNA Gel Extraction Kit from Sangon Biotech (Shanghai) Co., Ltd., Shanghai, China, followed by ligation into the pUCm-T (Sangon, Shanghai, China) Vector supplied by the same manufacturer. The conjugated product was then used to be transformed into *E. coli* DH5α cells (Takara, Dalian, China), followed by the extraction of the recombinant plasmid, which was submitted to Sangon Biotech (Shanghai, China) Co., Ltd. for sequencing.

### 2.4. Whole-Genome Sequence Splicing and Gene Analysis

The SeqMan module within the DNASTAR platform (version 11.1.0) facilitated fragment assembly, sequence alignment, and editing procedures using standard parameter settings. Full-genome sequences of GETV, collected from various regions and species, were downloaded from the GenBank database (accession numbers provided in Supplementary Table S3). Phylogenetic analysis was performed using MEGA software (version 7.0) with the maximum likelihood method based on the Kimura 2-parameter model, and bootstrap values were calculated from 1000 replicates. Sequence homology analysis was conducted using MegAlign software (version 10.2.6, DNASTAR) with the ClustalW algorithm and default alignment parameters.

## 3. Results

### 3.1. Sample Testing and Viral Isolation

**Figure 2:**
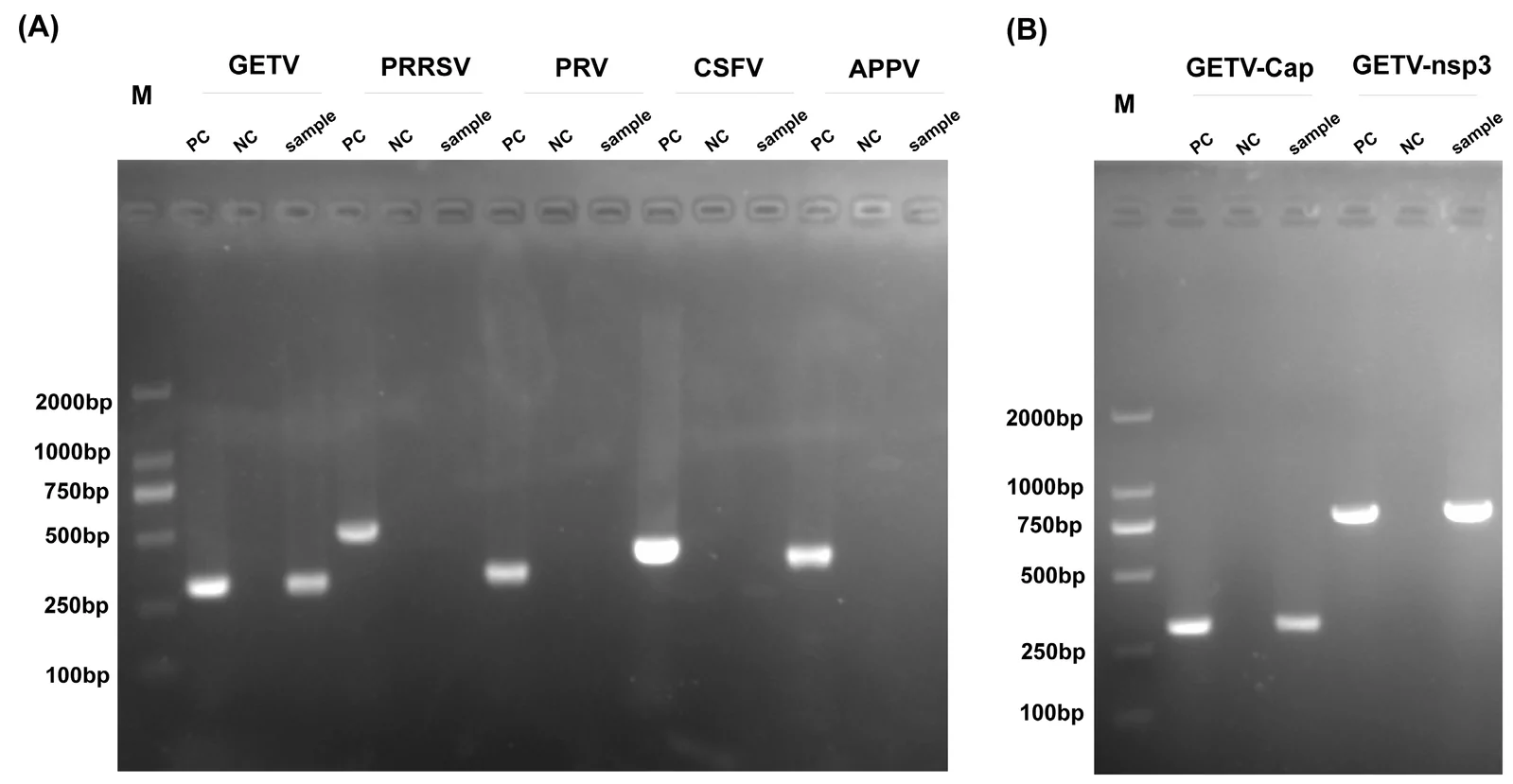
Agarose gel electrophoresis. (**A**) sample detection: M: DL2000 marker; PC: positive control; NC: negative control; Sample: clinical sample. GETV *Cap* (316 bp), positive in the isolate; PRRSV (514 bp), PRV (348 bp), CSFV (449 bp), and APPV (408 bp), negative. (**B**) Clinical sample infection in BHK-21 cells: M: DL2000 marker; PC: positive control; NC: negative control; Sample: BHK-21 cell supernatant. GETV *Cap* (316 bp) and GETV *nsp3* (810 bp), positive, indicating the presence of GETV in the BHK-21 cell supernatant.

In this study, samples were collected for pathogen detection from pigs exhibiting clinical symptoms such as locomotor dysfunction, neurological signs, mild diarrhea, and fatal outcomes in severe cases. These samples included spleen, lungs, and kidneys, as well as placental tissue and umbilical cord blood from aborted fetuses. GETV infection was confirmed by PCR(RT-PCR). The PCR(RT-PCR) results demonstrated that GETV genomic RNA was detected in the spleen tissues of infected pigs, while diagnostic tests for other common swine pathogens including PRV, PRRSV, CSFV, and APPV yielded negative results [30] (Figure 2A). Subsequently, positive spleen samples were used to inoculate BHK-21 cells. Following three consecutive rounds of passages, RNA extracted from these cultures tested positive for GETV using primers specific for the GETV *Cap* gene and *nsp3* gene (Figure 2B). This GETV isolate was designated as SC202009.

### 3.2. Viral Growth Dynamics and Cytopathic Effects

**Figure 3:**
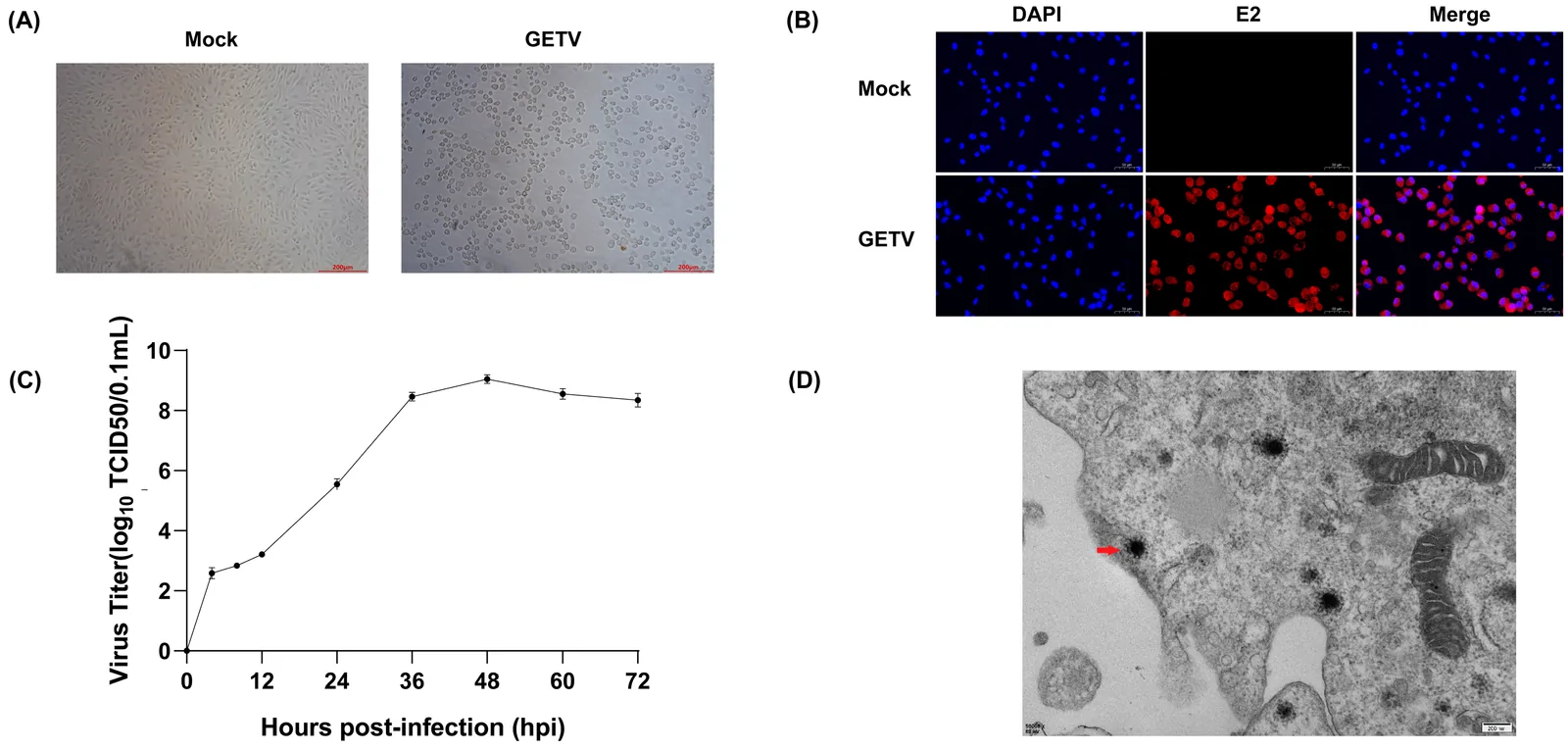
Infection and proliferation of the SC202009 strain in BHK-21 cells. (**A**) CPEs in BHK-21 cells were observed at 12 hpi with a scale bar of 200 µm. (**B**) Immunofluorescence analysis revealed the GETV E2 protein expression (visualized as red signals) within infected BHK-21 cells at 24 hpi, while nuclear regions appeared blue through DAPI counter-staining (scale bar: 50 µm). (**C**) Growth curve of the SC202009 strain in BHK-21 cells. Viral supernatants were collected from 0 to 72 hpi and titrated using the TCID_50_ method. (**D**) Electron micrographs of SC202009 virions observed by TEM (scale bar: 200 nm), with the red arrow pointing to the SC202009 virions. All values are presented as mean ± SD from three independent experiments.

At 12 h after inoculating the BHK-21 cells with GETV-positive samples, cultures were evaluated for cytopathic effects. Microscopic examination revealed that infected cells were shrunken, rounded, and detached, such that they were floating in some instances (Figure 3A). In indirect fluorescence immunoassays, GETV-infected cells treated with polyclonal rabbit anti-GETV E2 antibodies exhibited a pronounced red fluorescent signal (Figure 3B). The virus TCID_50_ measured on BHK-21 cells was 10^−9.125^/0.1 mL. A one-step growth curve analysis revealed that the maximum viral titer was evident at 48 h post-infection (hpi), after which it decreased slightly (Figure 3C). Transmission electron microscopy (TEM) images revealed that the virions exhibited a spherical morphology and were localized near the cell membrane within the interstitium (Figure 3D).

### 3.3. The SC202009 Genome

The complete SC202009 genome is 11,689 bp in length, with a nucleotide composition of 28.03% adenine (A), 26.17% guanine (G), 19.63% thymine (T), and 26.16% cytosine (C), resulting in an overall GC content of 52.33%. This genome encompasses five untranslated regions (UTRs), including one 3′ UTR, and two open reading frames (ORFs). The full SC202009 genome sequence has been uploaded to GenBank under accession number OK423758. The SC202009 strain of the virus was sequenced at the F1, F5, F10, F15, and F21 regions. The sequencing results demonstrated 100% genetic identity among these regions of the SC202009 strain, with no mutations detected.

### 3.4. Gene Sequence Analyses of SC202009

**Figure 4:**
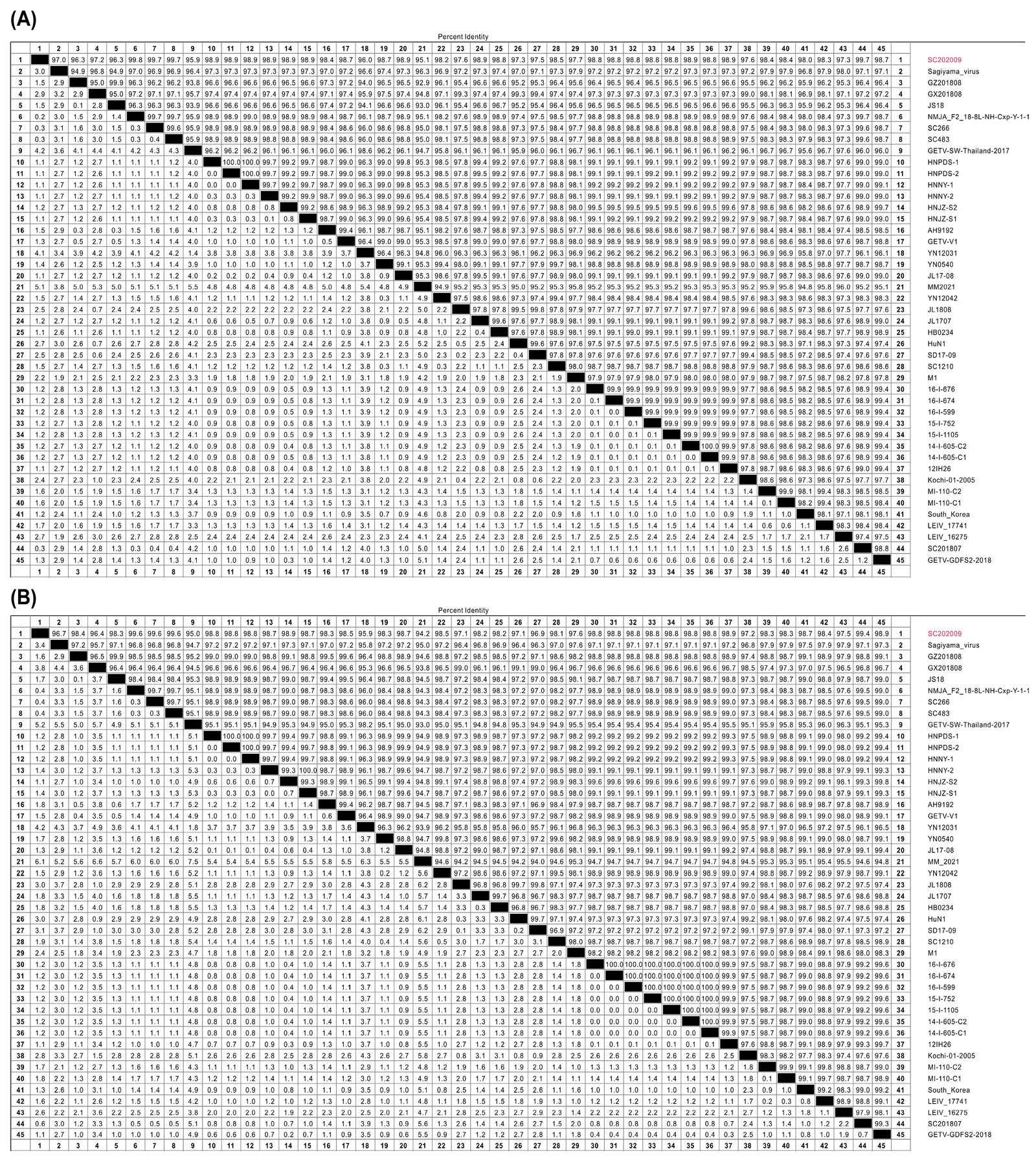
Genomic Homology Analysis of SC202009. (**A**) Complete genome homology analysis. (**B**) Homology analysis of the *E2* gene.The red letters designates the newly characterized SC202009 isolate.

When compared with reference sequences from 44 viral strains, the sequence homology for SC202009 ranged from 95.1% to 99.8%. Notably, it demonstrated a remarkable 99.8% homology with NMJA_F2_18-8L-NH-Cxp-Y-1-1, which was isolated from *Culex pipiens* in China. Additionally, this virus exhibited 99.7% homology with the SC266, SC483, and SC201807 strains, all of which were isolated from pigs in Sichuan. In contrast, it showed the lowest homology with the Hainan M1 strain, and shared 97.4% identity with HuN1, a strain isolated in Hunan Province in 2017 (Figure 4A). The *E2* gene sequence of SC202009 displayed a homology range of 94.2% to 99.6% with the available reference sequences. This included 99.6% homology with NMJA_F2_18-8L-NH-Cxp-Y-1-1, SC266, and SC483 Sichuan isolates and 97.6% homology with the M1 Hainan isolates. Conversely, it exhibited the lowest homology of 94.2% with the original MM2021 strain from Malaysia (Figure 4B).

**Figure 5:**
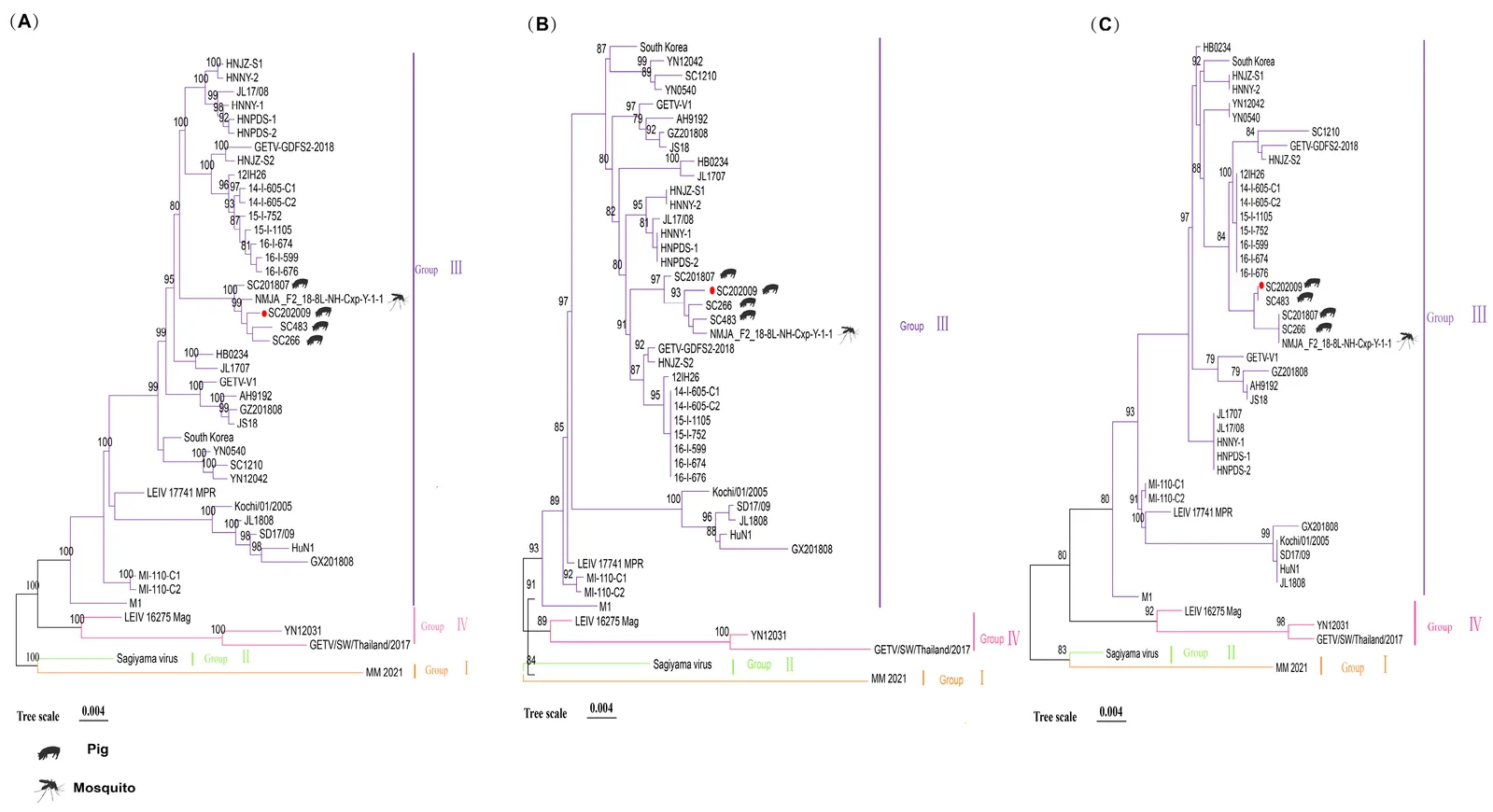
Phylogenetic analysis of the SC202009 strain and phylogenetic tree constructed using reference sequences to determine its genetic lineage [32,33]. (**A**) Phylogenetic tree based on the whole genome. (**B**) Phylogenetic tree based on the *E2* genome. (**C**) Phylogenetic tree based on the *Cap* genome. A filled crimson circle designates the newly characterized SC202009 isolate. Phylogenetic trees were constructed using the neighbor-joining method implemented in MEGA software (version 7.0), with 1000 bootstrap replications to assess the statistical reliability of the branching patterns. The tree scale is set to 0.004, indicating genetic distance. Different colors are used to differentiate lineages and reference strains, including those sourced from GenBank sequences.

Based on gene sequences, GETV isolates can be classified into four genotypes. The nucleotide sequences for the full GETV genome (Figure 5A), the *E2* gene (Figure 5B), and the *Cap* gene (Figure 5C) were used to establish three phylogenetic trees. SC202009 was classified into group GIII in these phylogenetic trees and clustered in the same branch as SC202018, SC266, SC483, and NMJA_F2_18-8L-NH-Cxp-Y-1-1.

**Figure 6:**
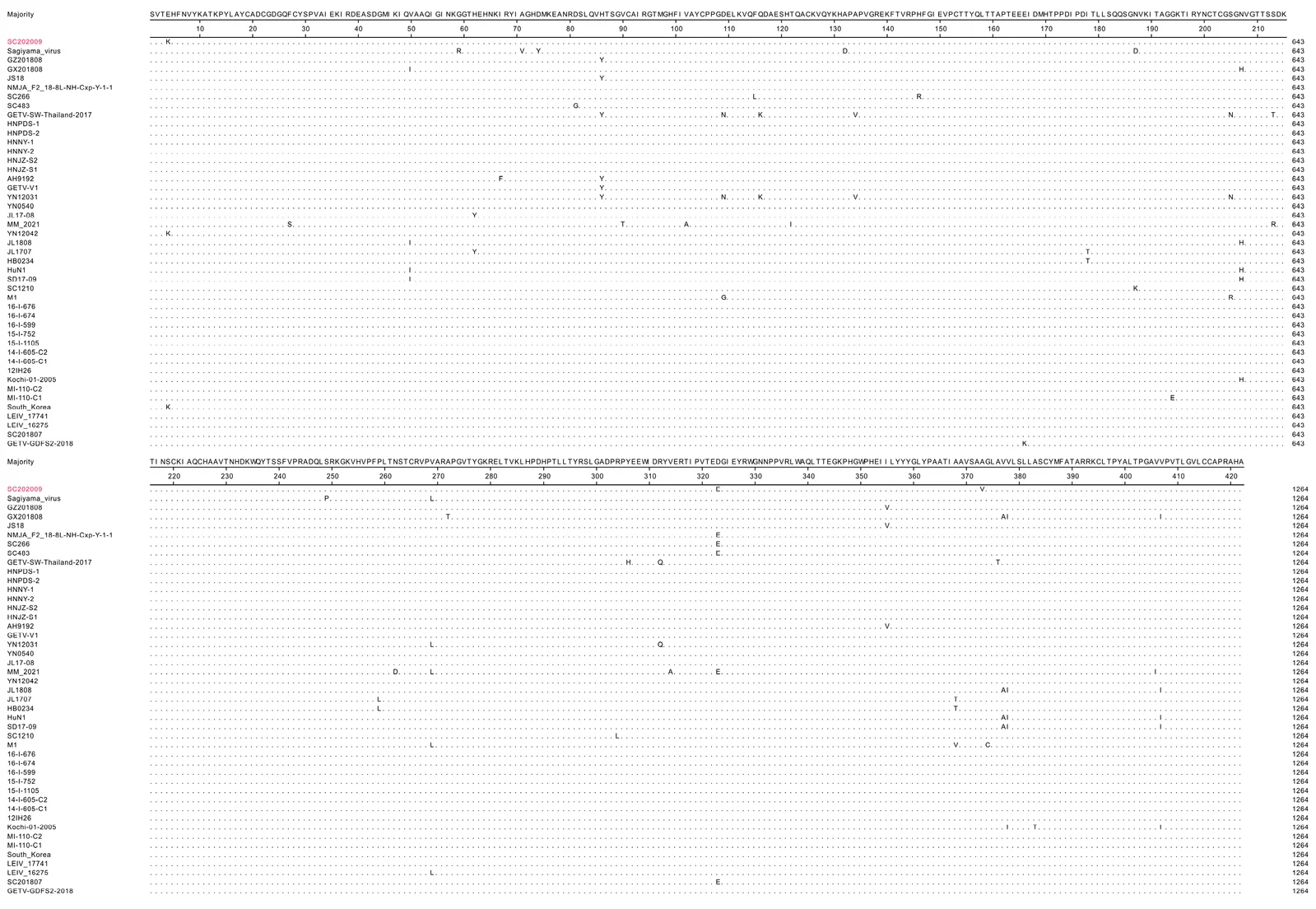
E2 protein amino acid sequence conservation analysis. The first sequence, labeled as ‘majority’, represents the most frequently occurring amino acid across all sequences, as identified using MEGA software (version 7.0). The red letters indicate the newly characterized SC202009 isolate, while variations in letters denote mutation sites within the amino acid sequence.

The GETV SC202009 *E2* gene is 1266 bp in length, encoding a 422-amino-acid protein. Compared to the reference E2 amino acid sequence, SC202009 demonstrates a sequence conservation of 97.2% to 99.5%, exhibiting significant homology with the E2 proteins of SC201807 and NMJA_F2_18-8L-NH-Cxp-Y-1-1. The only detected mutations were present at the 4th (Glu-Lys) and 373rd (Ala-Val) positions. Relative to the original Malaysian strain, SC202009 exhibits amino acid changes at the 27th (Ser-Phe), 90th (Thr-Val), 102nd (Ala-Val), 122nd (Ile-Thr), 213th (Arg-Ser), 262nd (Asp-Asn), 269th (Leu-Val), 314th (Ala-Val), and 406th (Ile-Val) positions (Figure 6).

## 4. Discussion

The epidemiological profile of GETV across Chinese territories has escalated in severity during recent periods, presenting significant risks to both veterinary and human health sectors. Early surveillance identified GETV presence in multiple mosquito vectors that were previously unreported. Vector competence studies have established mosquitoes belonging to *Aedes* and *Culex* genera as the principal vectors facilitating GETV transmission [4]. Initial GETV isolation in China occurred in 1964 from field-collected Culex specimens in Hainan Province. These arthropod vectors exhibit extensive geographical distribution throughout China and sustain substantial population densities. GETV exhibits broad geographical distribution across multiple regions (Figure 1). In this study, the SC202009 strain was isolated from porcine spleen tissue. Phylogenetic tree analysis revealed that it shared 99.8% homology with the NMJA_F2_18-8L-NH-Cxp-Y-1-1 strain (isolated from Inner Mongolia), which was isolated from Culex pipiens in China. Considering that GETV can be transmitted by mosquitoes across different species [33], it is plausible that SC202009 was introduced to Sichuan Province via mosquito vectors from northern regions of China. Alternatively, the virus may be spreading through the pig–mosquito–pig cycle, which could expand the geographical range of viral transmission. As China’s livestock industry expands and the trade and movement of livestock increase, the prevalence of vectors such as mosquitoes has also risen. To effectively control the spread of GETV, comprehensive integrated measures are recommended, including enhanced vector control strategies, improved farm biosecurity practices, the implementation of an all-in/all-out production system, the development of efficacious vaccines, and the establishment of real-time molecular surveillance networks.

GETV originated approximately 145 years ago and gradually evolved into four genomic groups (GI, GII, GIII, GIV) [2,14]. Group I comprises the prototype strain MM2021 exclusively, originally recovered from Malaysian mosquito populations. The SAGV isolate, characterized in Japan over six decades prior, represents a defining member of Group III [34]. Group IV represents a more recent evolutionary lineage that emerged approximately 30 years ago, with YN12031 serving as the representative strain. The majority of GETV strains circulating globally belong to Group III. Notably, genomic surveillance has revealed that every GETV isolate recovered from swine populations thus far demonstrates a genetic affiliation with Group III.

In this study, the GETV strain SC202009 was successfully isolated from porcine samples. The genome length of SC202009 was 11,689 bp. Comprehensive phylogenetic analyses of the full-length genome, *E2* gene, and *Cap* gene consistently classified SC202009 within Group GIII. SC202009 exhibited 95.1% to 99.8% homology with other subtypes, and 99.7% to 99.8% homology within the same evolutionary branch. Specifically, SC202009 showed 99.7% homology with three other Sichuan isolates (SC266, SC483, and SC201807), all of which were obtained from pigs. These results indicate a high level of genomic conservation among porcine GETV strains, suggesting the circulation of a dominant strain in Sichuan. As a major pig farming province, Sichuan requires enhanced GETV surveillance systems and the implementation of targeted prevention and control strategies. SC202009 was identified as the primary causative agent of the outbreak, confirmed by RT-PCR/PCR detection of GETV infection and the exclusion of other common swine viral pathogens (PRV, PRRSV, CSFV, and APPV). In contrast, the previously reported SC201807 strain, also isolated in Sichuan Province, was derived from serum samples that were co-infected with PRRSV and GETV, indicating a mixed infection scenario. Therefore, SC202009 serves as a more representative isolate for studying GETV pathogenesis, characterizing the predominant strain responsible for the pure GETV outbreaks in Sichuan Province from 2020 to 2021. The clinical manifestations associated with SC202009 included motor disorders, neurological symptoms, and mild diarrhea, with severe cases leading to mortality. This clinical presentation differs from that of previously documented GETV infections in pigs, which were typically characterized by dysgenesis, severe diarrhea, and high mortality. It also differs from that of the 2017 GETV outbreak in Hunan Province, which caused piglet mortality and reproductive disorders in over 150 pregnant sows [3]. These findings highlight the diversity of GETV-related symptoms, underscoring the importance of studying the pathogenicity and clinical manifestations of different GETV strains.

Viral morphogenesis and infectivity processes critically depend upon GETV structural proteins, including capsid, E3, E2, 6K, and E1 components. Among these, E1 and E2 are transmembrane proteins, with E1 primarily mediating membrane fusion during viral entry [35]. E2, a critical antigen, plays a central role in binding to host cell receptors, facilitating viral infection, disease progression, and the activation of immune responses. As the primary antigenic site for recognition and neutralization, E2 is crucial for viral adsorption, host infection, and the induction of antiviral immunity.

The E2 protein of the SC202009 strain exhibits high conservation, with only two mutations compared to the SC201807 strain. This conservation suggests functional similarity with other GETV strains, potentially contributing to its infectivity, immune evasion, and stability. As a surface glycoprotein critical for viral entry and immune recognition, the structural and functional consistency of E2 across GETV strains makes it an ideal target for vaccine development. With its conserved E2 protein and genetic stability, the SC202009 strain is a promising candidate for a broad-spectrum vaccine capable of protecting against diverse GETV strains, including emerging variants. Comprehensive genomic and functional analyses of the SC202009 strain can provide deeper insights into E2 protein immunogenicity, viral pathogenicity mechanisms, and vaccine-induced protective immune responses, thereby facilitating rational vaccine design, optimization, and eventual clinical application. In summary, the SC202009 strain not only serves as a valuable resource for studying GETV biology but also offers strong support for the development of effective vaccines.

Since the current study was conducted on a pig farm affected by an outbreak in Sichuan Province, further studies should investigate the clinical features, pathogenicity, and epidemiological characteristics of GETV in Sichuan Province.

## 5. Conclusions

Our results suggest that a dominant strain of GETV is circulating among pigs in Sichuan, with SC202009 showing 99.7–99.8% homology to previously isolated strains from the region. This indicates the presence of endemic GETV strains in Sichuan’s pig population.

## Data Availability

All datasets underlying our research results can be found within the main text and accompanying Supplementary Materials. The genome sequence of the GETV SC202009 isolate has been deposited into the GenBank database with the accession number MK693225.

## Supplementary Materials

The following supporting information can be downloaded at https://www.mdpi.com/article/10.3390/vetsci12030276/s1: Table S1: Primer sequence; Table S2: Primers for amplification of all sequences of genes of GETV; Table S3: GETV reference sequence information.
