# Supplementary material for: Isolation and Characterization of a Porcine Getah Virus Strain from Sichuan Province
Source: Vet Sci. 2025 Mar 15;12(3):276. doi: 10.3390/vetsci12030276 (PMC11945399; doi:10.3390/vetsci12030276)
Supplement: Supplementary file 1 [file vetsci-12-00276-s001.zip › vetsci-3471478-supplementary.pdf]

Supplementary Table

Supplementary Table S1

Table 1 Primer Sequence

Primer Name	Primer Sequence	Length/bp
PRV-F	GCTCTGCGTGCTTGTGCTCC	348
PRV-R	GGGTCCATTCGTCACTTCCG	
PRRSV-F	TTGATTGGGATGTTGTGCTTCCTGG	514
PRRSV-R	CGCGGAGCAGGGACAGGC	
CSFV-F	GACACTAGYGCAGGCAAYAG	449
CSFV-R	AGTGGGTTCCAGGARTACAT	
APPV-F	TGGGCGATGCGGTAATAGAG	408
APPV-R	AATAATTCGCTGCAGGTTGGA	
GETV-Cap-F	ACCGAAGAAGCCGAAGAAAAAGC	316
GETV-Cap-R	GCACTCCAGGTCATACTTGCTC	
GETV-Nsp3-F	CTTTCAGAGAGGACAGAGCGTACTC	810
GETV-Nsp3-R	CTCTTCTGCCGTTATCAAAGTTAGAAAACAG	

Supplementary Table S2

Table S2 Primers for amplification of all sequences of genes of GETV

Primer Name	Primer Sequence	Length/bp
1-F	TGACATCACCGTTCGCTCTTTCT	982
1-R	GGGTAACTGCGTAATCGACTGTT	
2-F	TCCACCTTGTACACCGAGAGC	964
2-R	TTTGGGGGGAAAGGATCAAGT	
3-F	GCGGCAGAAGAAGAAGAGAAGGA	924
3-R	TAGCAAAGTACCCGAGTGACACG	
4-F	GTGGCCAGTGGAAAGAAGGAGAA	862
4-R	GTATTCCGGCCGTCTCAAGAACT	
5-F	GTGACCCGTGGATAAAGGTGT	936
5-R	GGGCTGTCACCACCATCTCGC	
6-F	ACTGAGTACCGGCAACACCACTA	1093
6-R	AATCTGCGTCCTCTACTGGGCAT	
7-F	GGCGGTCGACATGGCTGAAAT	904
7-R	ACCGAACTGTATTCCTGTGCT	
8-F	ACCTAGACATCCAATTCGGTGAC	1034
8-R	GGTATGCTTGGTGCCTGGTGTAA	
9-F	GTGCCAATGGACCGCTTCGTGAT	997
9-R	TCTATTTAGGACCGCCGTACAGA	
10-F	AGGCTGTTCAAACTCGGGAA	1035
10-R	CAGTCCTAGCTCCTTCGTTGGC	
11-F	AGTGCAGTACAGCGGTGGTAGG	905
11-R	GCATGTCGATCTCTTCCTCCGT	
12-F	CCAGGGGACGAACTAAAGGT	1036
12-R	TTATGGCAGCGATCGGCGTT	
13-F	ACAACTGAAGGCAAACCCCATG	1123
13-R	AAGGCACGTGAACAGTACCGGA	

Supplementary Table S3

Table S3 GETV reference sequence information

Strain Name	GenBank Accession Number	Collection Year	Species
Kochi/01/2005	AB859822.1	2005	Sus scrofa
GETV-GDFS2-2018	MT086508.1	2018	Pig
SC201807	MK693225.1	2018	pig
LEIV 16275 Mag	EF631998.1	2007	Aedes sp.
LEIV 17741 MPR	EF631999.1	2007	Culex sp.
South Korea	AY702913.1	2004	Pig
MI-110-C1	LC079086.1	2016	Equus caballus
MI-110-C2	LC079087.1	2016	Equus caballus
12IH26	LC152056.1	2012	Culex tritaeniorhynchus
14-I-605-C1	LC079088.1	2014	Equus caballus
14-I-605-C2	LC079089.1	2014	Equus caballus
15-I-1105	LC212973.1	2015	Sus scrofa domesticus
15-I-752	LC212972.1	2015	Equus caballus
16-I-599	LC223130.1	2016	Equus caballus
16-I-674	LC223131.1	2016	Equus caballus
16-I-676	LC223132.1	2016	Equus caballus
M1	EU015061.1	2008	Culex sp.
SC1210	LC107870.1	2012	Armigeres subalbatus
SD17/09	MH106780.1	2017	fox
HuN1	MF741771.1	2017	Pig
HB0234	EU015062.1	2008	Culex tritaeniorhynchus
JL1707	MH722255.1	2017	mosquito
JL1808	MH722256.1	2018	cattle
YN12042	KY450683.1	2012	Culex tritaeniorhynchus
MM 2021	MN849355.1	1955	Culex gelidus
JL17/08	MG869691.1	2017	mosquito
YN0540	EU015063.1	2007	Armigeres subalbatus
YN12031	KY434327.1	2012	Armigeres subalbatus
GETV-V1	KY399029.1	2016	Pig
AH9192	MG865965.1	2017	Pig
HNJZ-S1	KY363862.1	2011	Pig
HNJZ-S2	KY363863.1	2015	Pig
HNNY-2	MG865967.1	2016	Pig
HNNY-1	MG865966.1	2016	Pig
HNPDS-1	MG865968.1	2017	Pig

Strain Name	GenBank Accession Number	Collection Year	Species
HNPDS-2	MG865969.1	2017	Pig
SC483	MN478486.1	2018	Pig
GX201808	MT269657.1	2018	Pig
GETV/SW/Thailand/2017	LC534253.1	2017	Sus scrofa
SC266	MN478487.1	2018	Pig
GZ201808	MK487997.1	2018	horse
NMJA _F2_18-8L-NH-Cxp-Y-1-1	MW246769.1	2018	Culex pipiens
JS18	MT210319.1	2018	Pig
Sagiyama virus	AB032553.1	1999	
